# Comparative analysis of the secretomes of *Schizophyllum commune* and other wood-decay basidiomycetes during solid-state fermentation reveals its unique lignocellulose-degrading enzyme system

**DOI:** 10.1186/s13068-016-0461-x

**Published:** 2016-02-20

**Authors:** Ning Zhu, Jiawen Liu, Jinshui Yang, Yujian Lin, Yi Yang, Lei Ji, Meng Li, Hongli Yuan

**Affiliations:** State Key Laboratory of Agrobiotechnology, College of Biological Sciences, China Agricultural University, 100193 Beijing, China; National Energy R&D Center for Non-food Biomass, China Agricultural University, 100193 Beijing, China

**Keywords:** *Schizophyllum commune*, Solid-state fermentation, White rot, Brown rot, Polysaccharide-degrading enzyme, Secretome, Hydroxyl radical, LPMO, Expansin

## Abstract

**Background:**

The genome of *Schizophyllum commune* encodes a diverse repertoire of degradative enzymes for plant cell wall breakdown. Recent comparative genomics study suggests that this wood decayer likely has a mode of biodegradation distinct from the well-established white-rot/brown-rot models. However, much about the extracellular enzyme system secreted by *S. commune* during lignocellulose deconstruction remains unknown and the underlying mechanism is poorly understood. In this study, extracellular proteins of *S. commune* colonizing Jerusalem artichoke stalk were analyzed and compared with those of two white-rot fungi *Phanerochaete chrysosporium* and *Ceriporiopsis subvermispora* and a brown-rot fungus *Gloeophyllum trabeum*.

**Results:**

Under solid-state fermentation (SSF) conditions, *S. commune* displayed considerably higher levels of hydrolytic enzyme activities in comparison with those of *P. chrysosporium, C. subvermispora* and *G. trabeum*. During biodegradation process, this fungus modified the lignin polymer in a way which was consistent with a hydroxyl radical attack, similar to that of *G. trabeum*. The crude enzyme cocktail derived from *S. commune* demonstrated superior performance over a commercial enzyme preparation from *Trichoderma longibrachiatum* in the hydrolysis of pretreated lignocellulosic biomass at low enzyme loadings. Secretomic analysis revealed that compared with three other fungi, this species produced a higher diversity of carbohydrate-degrading enzymes, especially hemicellulases and pectinases acting on polysaccharide backbones and side chains, and a larger set of enzymes potentially supporting the generation of hydroxyl radicals. In addition, multiple non-hydrolytic proteins implicated in enhancing polysaccharide accessibility were identified in the *S. commune* secretome, including lytic polysaccharide monooxygenases (LPMOs) and expansin-like proteins.

**Conclusions:**

Plant lignocellulose degradation by *S. commune* involves a hydroxyl radical-mediated mechanism for lignocellulose modification in parallel with the synergistic system of various polysaccharide-degrading enzymes. Furthermore, the complex enzyme system of *S. commune* holds significant potential for application in biomass saccharification. These discoveries will help unveil the diversity of natural lignocellulose-degrading mechanisms, and advance the design of more efficient enzyme mixtures for the deconstruction of lignocellulosic feedstocks.

**Electronic supplementary material:**

The online version of this article (doi:10.1186/s13068-016-0461-x) contains supplementary material, which is available to authorized users.

## Background

Lignocellulosic feedstock from plant materials is considered to be an abundant renewable resource with increasing potential for the production of alternative liquid fuels. One of the main challenges for large-scale production of economically competitive bioethanol has been the high cost of enzymatic hydrolysis process [1]. Because conversion of biomass polysaccharides into fermentable sugars depends exclusively on the efficiency of enzyme mixtures [2], optimizing the mixture composition could contribute to the hydrolysis efficiency and reduction of enzyme loadings. Many fungi and bacteria are capable of utilizing cellulose and hemicellulose through the secretion of polysaccharide-degrading enzymes. The filamentous fungi *Trichoderma reesei* has been widely exploited in industrial applications for the production of commercial cellulases [3]. However, the enzyme pool produced by *T. reesei* via submerged fermentation is deficient on certain components and requires additional enzyme supplement for efficient and complete hydrolysis of complex lignocellulosic materials [4, 5]. For this reason, considerable research efforts have been devoted to exploring alternative fungi for the production of cost-effective enzymes and development of more efficient enzyme cocktails [6–8].

*Schizophyllum commune* is a widely distributed saprophytic basidiomycete around the world and its genome has been previously sequenced. The carbohydrate-active enzyme database (CAZy) annotation of *S. commune* identifies a total of 366 carbohydrate-active enzymes (CAZymes), of which 106 are predicted to be involved in plant polysaccharide degradation [9]. Compared to *T. reesei*, *S. commune* owns an equivalent number of members from GH families acting on cellulose, including GH5, 6, 7, 12, and 45. Analysis of the diversity of non-cellulosic polysaccharide-degrading enzymes showed that *S. commune* has more abundant xylan and pectin degradation-related glycoside hydrolases such as GH5, 10, 28, 43, 51, 53, 93, 105, and 115 families. In particular, the *S. commune* genome possesses a much larger number of GH43 proteins (19 in *S. commune* vs only 2 in *T. reesei*), indicating its great potential for hemicellulose and pectin deconstruction. In addition to hydrolytic enzymes, *S. commune* has an expanded complement of genes encoding lytic polysaccharide monooxygenases (LPMOs) of auxiliary activity (AA) family 9 (22 in *S. commune* vs 3 in *T. reesei*), which participate in oxidative cleavage of crystalline cellulose [10, 11]. Because the genome of *S. commune* encodes an extensive catalog of genes implicated in lignocellulose decomposition, its lignocellulolytic enzyme pool is expected to provide a prospective enzyme source for biotechnological applications. Several novel families of enzymes that remove xylan side chains, such as acetyl xylan esterases [12], glucuronoyl esterases [13], and α-glucuronidases [14, 15] have been identified in the cellulolytic system of *S. commune*. Among the few studies regarding its polysaccharide hydrolases, a recombinant endoxylanase from *S. commune* has been reported to exhibit very high level of activity against beechwood xylan [16], and crude enzymes containing mainly β-glucosidase from this fungal species could saccharify pretreated lignocellulose comparably well with a commercial β-glucosidase [17]. To date, studies of this species have focused mainly on the mating-type gene function [18, 19] and mushroom development [20, 21], whereas the lignocellulolytic enzymes produced by *S. commune* are far less characterized and a comprehensive study on its enzymatic system is still lacked.

Wood-degrading basidiomycetes have been typically classified as white-rot and brown-rot fungi according to the types of decay that they cause [22]. White-rot fungi such as *Phanerochaete chrysosporium* simultaneously break down all polymeric components of plant cell walls, including cellulose, hemicellulose, and lignin. Other white-rot species like *Ceriporiopsis subvermispora* selectively degrade lignin with little cellulose loss. In contrast, brown-rot fungi represented by *Gloeophyllum trabeum*, efficiently depolymerize the cellulose portion without substantial removal of lignin [23]. *S. commune* has been previously characterized as a white-rot species despite very limited lignin-degrading capacity [24]. With respect to lignin degradation, the genome of *S. commune* lacks genes encoding class II peroxidases of AA family 2, which is similar to brown-rot species [9]. Recent genomic comparisons of 33 basidiomycetes suggest that *S. commune* is an intermediate between white-rot and brown-rot species in terms of gene families encoding lignocellulose-degrading enzymes [25]. Given the limited reports characterizing its lignocellulolytic enzymes [26–28] and wood decay process [29], it is not clear what biodegradative strategy *S. commune* uses to decompose lignocellulosic biomass.

Jerusalem artichoke is one of the potential energy crops that can grow well on marginal lands in harsh environment [30]. This plant is considered as a promising candidate for consolidated bioprocessing (CBP), which enables the utilization of whole plant biomass (the tuber and stalk). Jerusalem artichoke tuber, with a high content of inulin, has been investigated as a sugar source for bioethanol production [31]. As for Jerusalem artichoke stalk, there is no report about using its lignocellulosic materials for fungal growth and induction of lignocellulolytic enzymes.

The aim of the present study is to identify potentially important enzymes and provide insight into the mechanism of plant cell wall deconstruction by *S. commune*. Here we report the degradation dynamics and lignocellulolytic enzyme pattern of *S. commune* cultivated on Jerusalem artichoke stalk during solid-state fermentation (SSF). This complex lignocellulosic substrate and the SSF culture conditions simulate more closely its decay process occurring in nature. The crude enzymes derived from *S. commune* were examined for the enzymatic saccharification of pretreated lignocellulosic biomass. The composition of extracellular protein profile of *S. commune* was identified by nano liquid chromatography-tandem mass spectrometry (nanoLC-MS/MS) and compared with those of representative white-rot and brown-rot basidiomycetes to gain a deeper understanding of the lignocellulose-degrading enzyme system of this fungus.

## Results

### Scanning electron microscopy (SEM)

**Fig. 1:**
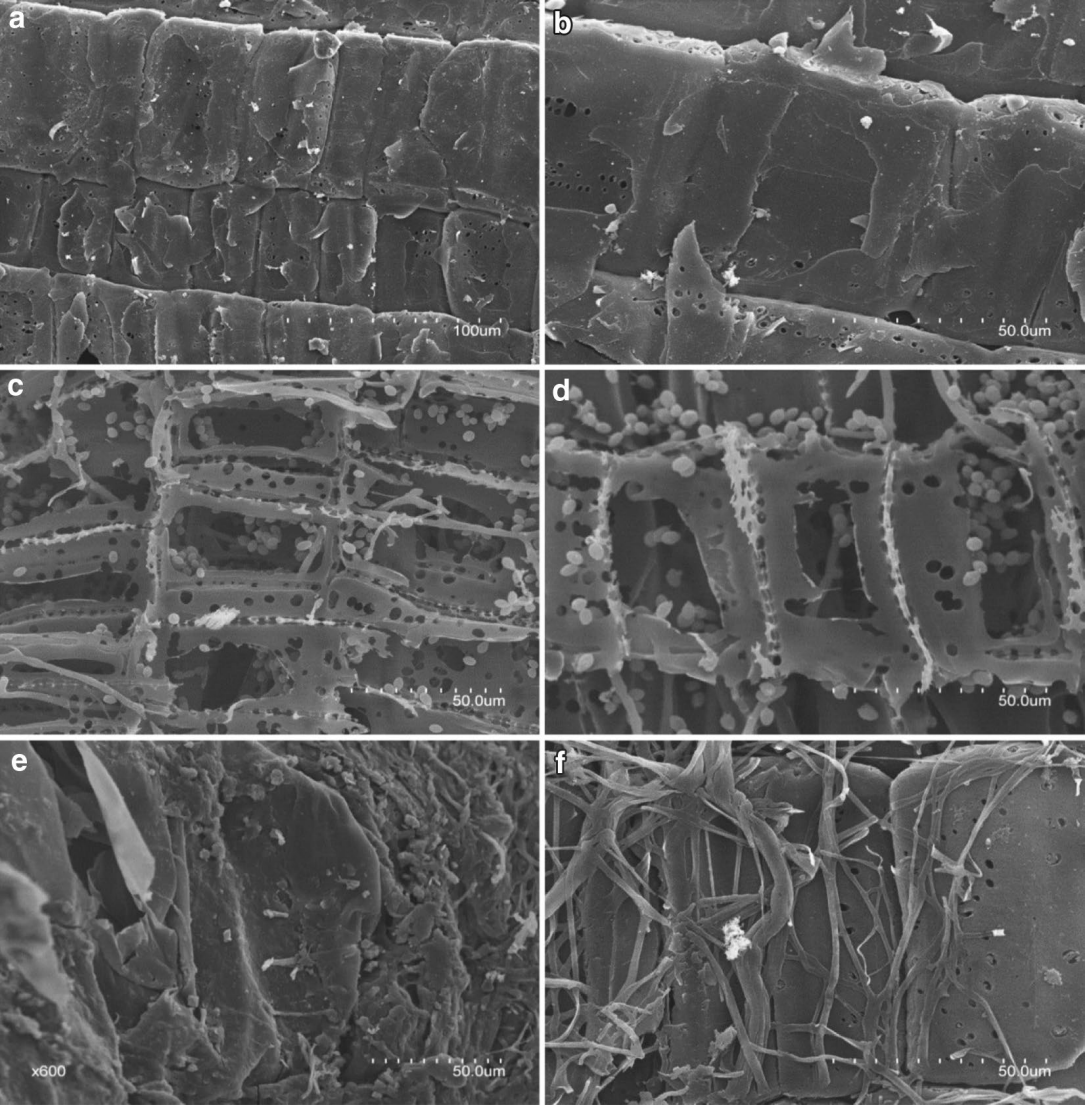
Scanning electron micrographs of Jerusalem artichoke stalk. **a**, **b** SEM image of Jerusalem artichoke stalk without any fungal inoculation. **c** SEM image of stalk sample inoculated with *P. chrysosporium* for 30 days. **d** SEM image of sample inoculated with *C. subvermispora* for 30 days. **e** SEM image of sample inoculated with *G. trabeum* for 30 days. **f** SEM image of sample inoculated with *S. commune* for 30 days

Scanning electron microscopy was applied to investigate the micromorphological characteristics of degraded Jerusalem artichoke stalk after inoculation with different fungal species. Figure 1a and b depicted non-decayed stalk cell walls with an intact morphology, while in stalk sample inoculated with *P. chrysosporium*, hyphae and spores were visible growing in the cavities formed by extensive decay, implying the degradation of all cell wall components (Fig. 1c). Sample subjected to inoculation with *C. subvermispora* exhibited a similar eroded morphology, but to a lesser extent (Fig. 1d). In contrast to white rot, stalk sample inoculated with *G. trabeum* was not completely degraded but appeared to have a distorted and collapsed structure because of more brittle and weakened cell walls (Fig. 1e). In stalk sample colonized by *S. commune*, the plant cell walls remained relatively intact despite the fungal mycelia covering the stalk surface (Fig. 1f). However, adjacent cells appeared to be separated from each other, probably due to degraded middle lamellae. Physical invasion of cell walls by fungal mycelia was also seen through the fissures between cells.

### Fungal degradation of Jerusalem artichoke stalk

Table 1 showed the degradation of the Jerusalem artichoke stalk components by four fungal species during SSF over an incubation period of 30 days. Incubation with *S. commune* for 30 days resulted in decrease in the cellulose and hemicellulose contents by 18.4 and 17.5 %, respectively, with a limited lignin degradation of 3.2 %. In comparison, *C. subvermispora* selectively degraded lignin as much as 45.4 %, while the cellulose content decreased by only 4.3 % in 30 days, indicating a preferential removal of lignin with respect to cellulose. And there was up to 22.9 % hemicellulose reduction along with lignin degradation, probably due to increasing accessibility of hemicellulose as lignin decomposed. At the end of cultivation, cellulose and hemicellulose loss caused by *P. chrysosporium* reached 33.3 and 31.6 %, respectively, the highest among four fungal species. A concomitant lignin degradation of 23.6 % was observed with the polysaccharide loss. Such simultaneous removal of all lignocellulosic components was consistent with the typical white rot. On the contrary, the brown-rot fungus *G. trabeum* caused a polysaccharide degradation of 12.2 % cellulose and 9.8 % hemicellulose, with a very minor lignin removal of 2.3 %.

**Table 1:** Degradation of Jerusalem artichoke stalk components by four fungi during solid-state fermentation

Days	*S. commune*			*P. chrysosporium*			*C. subvermispora*			*G. trabeum*		
	Loss (% initial content)			Loss (% initial content)			Loss (% initial content)			Loss (% initial content)		
	Cel	Hem	Lig	Cel	Hem	Lig	Cel	Hem	Lig	Cel	Hem	Lig
5	2.1 ± 0.7	1.2 ± 0.1	0.6 ± 0.1	6.9 ± 0.3	4.9 ± 0.4	3.5 ± 0.2	1.5 ± 0.2	3.2 ± 0.6	7.7 ± 0.7	1.5 ± 0.2	1.7 ± 0.3	0.5 ± 0.1
10	3.7 ± 0.5	3.1 ± 0.2	1.1 ± 0.3	12.3 ± 0.7	9.3 ± 0.6	8.1 ± 0.2	1.9 ± 0.2	6.0 ± 0.2	15.9 ± 0.2	3.0 ± 0.3	3.0 ± 0.1	0.8 ± 0.1
15	8.7 ± 0.3	6.4 ± 0.2	1.4 ± 0.1	19.4 ± 0.3	17.2 ± 0.7	11.9 ± 0.7	2.5 ± 0.8	10.5 ± 0.7	25.4 ± 0.6	4.9 ± 0.5	3.8 ± 0.2	1.2 ± 0.1
20	11.3 ± 0.8	9.7 ± 0.8	2.1 ± 0.3	25.5 ± 0.6	22.0 ± 0.5	15.4 ± 0.5	3.1 ± 0.4	15.5 ± 0.6	33.4 ± 0.8	8.4 ± 0.6	4.6 ± 0.4	1.7 ± 0.2
25	15.9 ± 0.2	13.3 ± 0.9	2.7 ± 0.4	30.1 ± 0.6	27.5 ± 1.1	21.1 ± 0.6	3.8 ± 0.7	19.0 ± 1.2	38.8 ± 1.5	10.1 ± 0.8	7.7 ± 0.5	2.1 ± 0.3
30	18.4 ± 0.9	17.5 ± 1.2	3.2 ± 0.4	33.3 ± 1.2	31.6 ± 0.5	23.6 ± 0.2	4.3 ± 0.3	22.9 ± 0.6	45.4 ± 1.1	12.2 ± 1.1	9.8 ± 0.9	2.3 ± 0.3

Cellulose (Cel), hemicellulose (Hem), and lignin (Lig) losses of Jerusalem artichoke stalk are presented for each fungal species. The data indicate mean values ± standard deviations from three replicates

### Pyrolysis gas chromatography-mass spectrometry (Py-GC/MS) analysis

Pyrolysis coupled with gas chromatography–mass spectrometry is a useful tool for characterizing decay patterns of wood-degrading fungi, especially chemical features of lignin structure [32]. During analysis of sound Jerusalem artichoke stalk and samples incubated with different fungal species, some differences were observed in the relative abundances of the released pyrolysis products (Table 2), indicating the divergent degradation patterns caused by the different fungi. The two white-rot fungi, *C. subvermispora* and *P. chrysosporium* preferentially degraded lignin moiety in stalk as evidenced by a decrease in the ratio of lignin- to carbohydrate-derived pyrolysates (lignin/carbohydrate ratio). *G. trabeum* and *S. commune*, on the other hand, caused an increased ratio between lignin and carbohydrate pyrolysis products, revealing a preferential consumption of polysaccharides relative to lignin. Regardless of the decay patterns and substrate preferences, all four fungi seemed to demethoxylate syringyl units of lignin, thus decreasing the ratio of syringyl- to guaiacyl-type pyrolysates (S/G ratio).

**Table 2:** Relative peak areas (%) of lignin-derived pyrolysates identified after Py–GC/MS of fungi-rotting stalk

Compound	Control	*S. commune*	*P. chrysosporium*	*C. subvermispora*	*G. trabeum*
Guaiacol (G)	0.29	0.42	0.54	0.47	0.39
3-Methoxycatechol	nd^b^	0.32	nd	nd	0.43
4-vinylguaiacol	0.45	0.63	0.32	0.20	0.69
Syringol(S)	0.42	0.52	0.30	0.23	0.34
4-Methylsyringol	nd	0.15	nd	0.08	0.16
*Trans*-Isoeugenol	0.10	0.18	0.11	nd	0.09
4-Vinylsyringol	nd	0.17	0.12	nd	0.14
4-Allylsyringol	0.28	0.21	0.12	0.10	0.23
Carbohydrate	41.37	38.17	46.30	50.45	35.42
Syringyl/guaiacyl ratio	0.83	0.68	0.56	0.61	0.74
Pch-C1,2/Pch-C3 ratio^a^	1.18	3.26	1.91	2.80	3.09
Lignin/carbohydrate ratio	0.04	0.07	0.03	0.02	0.07

Jerusalem artichoke stalk without fungal inoculation is used as control

^a^ Ratio of phenylmethane and phenylethane to phenylpropane-type compounds

^b^ not detected

Although *G. trabeum* and *S. commune* preferentially degraded the carbohydrate polymers, both fungal species caused a significant increase in the ratio of phenylmethane and phenylethane units to phenylpropane units (Ph-C1,C2/Ph-C3 ratio), suggesting modification on the lignin moiety through extensive cleavage of side-chain linkages. A closer inspection of lignin-derived compounds showed that methoxyl groups linked to syringyl subunits were demethylated, as evidenced by the identification of 3-methoxycatechol pyrolysate. Such pyrolysis product was undetectable in the spectrum of sound stalk and samples decayed by *C. subvermispora* and *P. chrysosporium*.

### Determination of lignocellulolytic enzyme activities and iron-reducing capacity during SSF

**Fig. 2:**
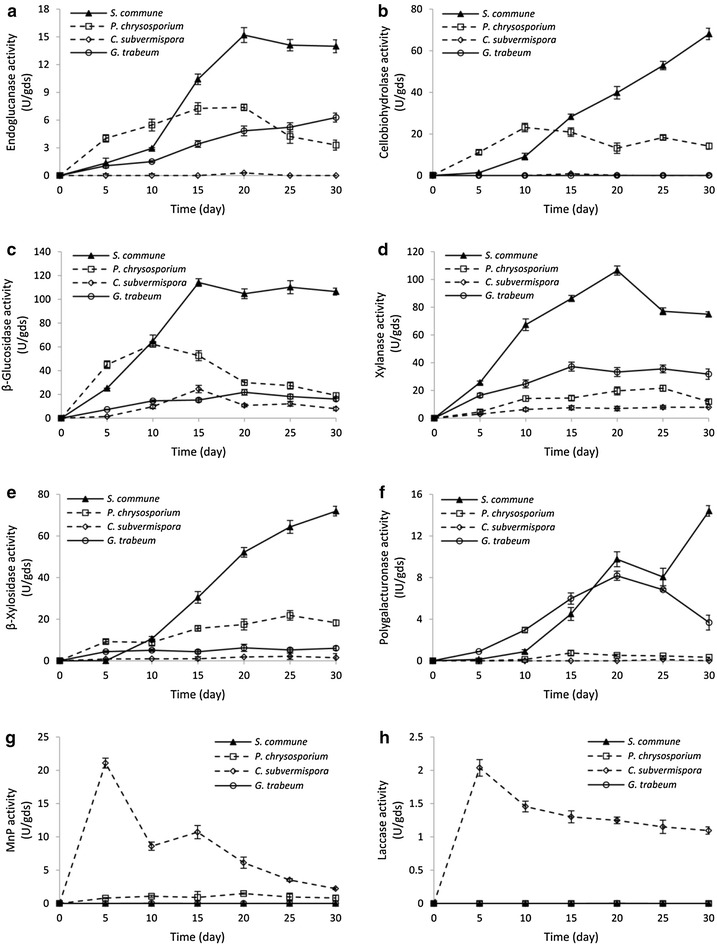
Lignocellulolytic enzyme activities in the secretomes of four fungi during SSF on Jerusalem artichoke stalk. The activities of cellulases (endoglucanase, cellobiohydrolase, and β-glucosidase) are shown in **a**, **b**, and **c**, respectively. The activities of hemicellulases (endoxylanase and β-xylosidase) are shown in **d** and **e**, respectively. The polygalacturonase activities are shown in **f**. The activities of ligninolytic enzymes (manganese peroxidase and laccase) are shown in **g** and **h**, respectively. The values shown are the mean of three replicates and the *error bars* indicate standard deviations from the mean values

The present study found that the four fungal species showed different time courses of lignocellulolytic enzyme activities in the extracellular extracts during growth on Jerusalem artichoke stalk (Fig. 2). In general, *S. commune* produced significantly higher levels of cellulolytic, xylanolytic, and pectinolytic activities compared to the other three fungi.

When comparing the activities of cellulases produced by the four fungal species, endoglucanase activity in *S. commune* extract increased gradually over time and reached a fairly constant level of around 15.2 ± 1.1 U/gds after day 20 (Fig. 2a). As for *P. chrysosporium*, the activity increased slowly to achieve maximum on day 20 (7.4 ± 1.2 U/gds), and declined afterward. Endoglucanase activity in *G. trabeum* extract increased over time (a maximum of 6.3 ± 1.5 U/gds on day 30), while it was negligible in *C. subvermispora*. Cellobiohydrolase activity in *S. commune* extract increased over the incubation time, and the highest activity (68.1 ± 2.2 U/gds) was obtained at the end of cultivation (Fig. 2b). In contrast, the maximum exoglucanase activity in *P. chrysosporium* extract (23.1 ± 3.0 U/gds) was obtained on day 10, and the activity was not detected or too low in *C. subvermispora* and *G. trabeum*(Fig. 1b). Beta-glucosidase activities were detected in all four fungi, but with different activity levels (Fig. 2c). In *S. commune* extract, the activity increased gradually to reach its highest level (114.1 ± 7.2 U/gds), which was more than twofold, fourfold, and fivefold as high as those of *P. chrysosporium* (62.2 ± 3.2 U/gds), *C. subvermispora* (24.5 ± 1.5 U/gds), and *G. trabeum* (21.8 ± 1.1 U/gds), respectively. Beta-glucosidase activities in the latter three fungal extracts increased at first and decreased subsequently.

Among hemicellulases, the xylanase activity of *S. commune* achieved a maximum of 106.5 ± 3.3 U/gds on day 20, after which it decreased to 44.9 ± 1.8 U/gds at the end of cultivation (Fig. 2d). The corresponding activity in *P. chrysosporium* increased over time and a peak (21.6 ± 1.4 U/gds) appeared on day 25. For *G. trabeum*, the activity increased to its highest value on day 15 (37.2 ± 2.5 U/gds) and then became relatively constant. Xylanase activity in *C. subvermispora* remained at a low level during the incubation. From the fifth day, β-xylosidase activity of *S. commune* increased gradually until the end of the incubation (72.0 ± 2.3 U/gds), while the corresponding activity produced by *P. chrysosporium* achieved a maximum of 21.8 ± 1.7 U/gds on day 25 (Fig. 2e). The activity in *G. trabeum* fluctuated at a level between 4.4 ± 0.3 and 6.3 ± 0.9 U/gds during the incubation period. *C. subvermispora* extract contained only a minor β-xylosidase activity.

As for pectinases, the maximum polygalacturonase activity of *S. commune* (14.4 ± 2.3 U/gds) was obtained at the end of incubation (Fig. 2f). As for *G. trabeum*, the polygalacturonase activity increased to a maximum of 8.2 ± 0.4 U/gds on day 20 and decreased markedly afterward. For comparison, the activity remained at a low level in *P. chrysosporium* extract in the incubation period and was not detected in *C. subvermispora* extract. Pectin lyase activity was below detectable level in all four fungal extracts (see Additional file 1: Figure S1).

In terms of ligninolytic enzymes, manganese peroxidase (MnP) from *C. subvermispora* showed high activity (21.1 ± 1.6 U/gds) on day 5 and the activity declined quickly with the incubation time (Fig. 2g). The MnP activity in *P. chrysosporium* extract maintained at a low level with a maximum activity of 1.5 ± 0.1 U/gds. Laccase (Lac) activity was detected only in *C. subvermispora* and showed a similar trend to MnP, but the peak activity was merely 2.0 ± 0.1 U/gds on the fifth day (Fig. 2g). A very low lignin peroxidase (LiP) activity was observed in *P. chrysosporium* extract, while no LiP activity was detected in *C. subvermispora* (see Additional file 1: Figure S1). In the case of *S. commune* and *G. trabeum*, no ligninolytic activities were detected in their culture extracts during the overall incubation period. For the selective ligninolytic fungus *C. subvermispora*, the ligninolytic enzymes (Lac and MnP) were produced from the beginning of cultivation and their activities decreased over time, while the activities of cellulases and hemicellulases were detected after 5 days of cultivation. Our results with enzyme activities are in accordance with recent secretomic analysis showing the temporal expression patterns of lignocellulytic enzymes of *C. subvermispora* in aspen-containing liquid medium [33].

**Fig. 3:**
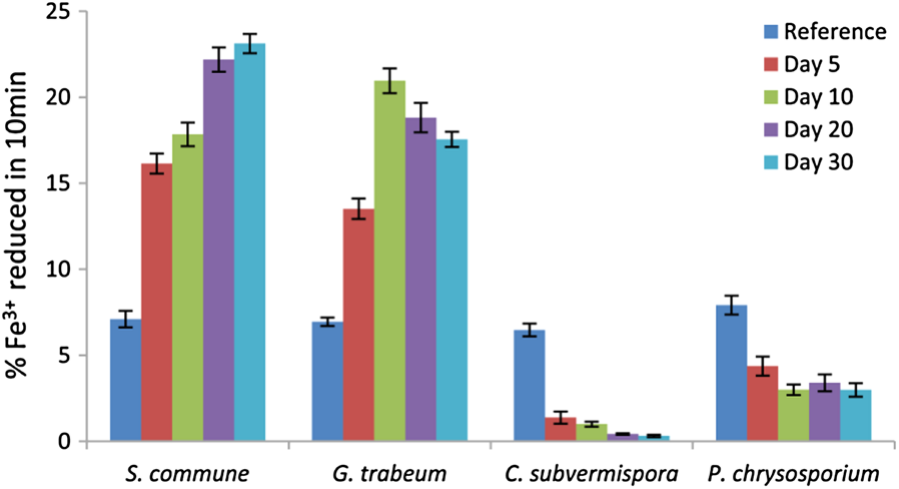
Iron-reducing capacities of the extracellular extracts produced by four fungi grown on Jerusalem artichoke stalk. Extracellular extracts are sampled after inoculation for 5, 10, 20, and 30 days, respectively. Extracts of Jerusalem artichoke stalk before fungal inoculation are used as reference. The data represent the mean of three replicates and the *error bars* indicate standard deviations from the mean values

For typical Fenton reactions, a system with the capability to potentiate the reduction of Fe^3+^ to Fe^2+^ is required. A remarkable difference was observed regarding the Fe^3+^-reducing activities in the fungal culture extracts (Fig. 3). Analysis of extracellular extracts recovered from *S. commune* showed that its Fe^3+^-reducing activity was constantly increased during the incubation period, suggesting the production of extracellular metabolites or enzymes capable of reducing iron ions. As for *G. trabeum*, the Fe^3+^-reducing activity increased rapidly in the first 10 days and then decreased slightly. It has been shown that *G. trabeum* can produce iron-reducing hydroquinones [34, 35] and low-molecular weight glycopeptides [36] for Fenton chemistry. *C. subvermispora*, on the other hand, showed a rapid decline of Fe^3+^-reducing activity in its extracellular extracts in the first 5 days of biodegradation, which was in agreement with previous report that hexadecylitaconic acids (known as ceriporic acid B) produced by *C. subvermispora* could inhibit the iron reduction [37]. *P. chrysosporium* also showed a similar trend of decreased Fe^3+^-reducing activity. This decrease of the Fe^3+^-reducing activity could be related to the consumption of extractives with Fe^3+^-reducing activity.

### Saccharification experiment

The extracellular enzyme system of *S. commune* was selected for the hydrolysis of lignocellulosic biomass since it produced the highest hydrolytic enzyme activities among four fungi in the study. Specific activities of the crude enzyme cocktail derived from *S. commune* were determined using model substrates and compared with those of a commercially available enzyme preparation from *Trichoderma longibrachiatum* (Table 3). The *S. commune* enzyme cocktail displayed comparable levels of cellulase activities to the commercial preparation. Specifically, enzymes from *S. commune* were slightly higher in endoglucanase and exoglucanase activities but lower in β-glucosidase activity. For hemicellulosic substrates, much higher levels of endoxylanase and β-xylosidase activities were present in the *S. commune* cocktail than the *T. longibrachiatum* preparation, indicating its high hydrolytic activity toward xylan. The two enzyme mixtures also showed difference in their specific activities against pectin. The *S. commune* enzymes exhibited more than twofold higher level of polygalacturonase activity relative to *T. longibrachiatum* enzymes.

**Table 3:** Comparison of specific activities (U/mg protein) of the two enzyme cocktails on model substrates

Enzyme source	CMC	PNPC	PNPG	Beechwood xylan	pNPX	Pectin
*S. commune*	21.5 ± 1.6	60.7 ± 2.8	108.8 ± 3.9	112.5 ± 2.3	55.6 ± 3.0	17.3 ± 1.6
*T. longibrachiatum*	19.7 ± 2.9	52.2 ± 1.9	129.0 ± 2.7	26.6 ± 3.2	34.8 ± 1.4	5.2 ± 1.3

**Fig. 4:**
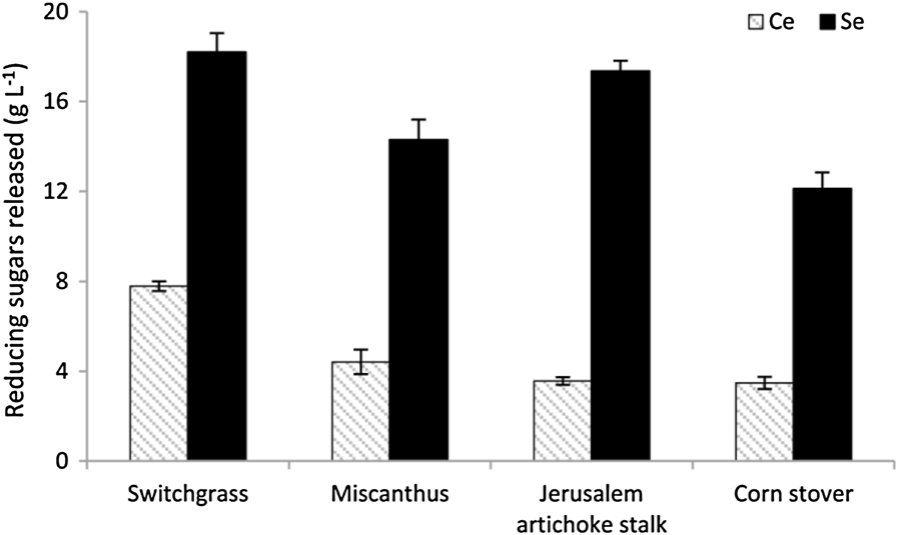
Release of reducing sugars from pretreated lignocellulosic biomass by *S. commune* enzymes and commercial enzymes. The hydrolysis reactions were performed at 37 °C for 24 h incubation with 2 % (w/v) biomass loading. Enzyme loading was set at 20 mg protein/g glucan. The amount of reducing sugars released was determined using the DNS method. *Se* *S. commune* enzyme cocktail, *Ce* commercial enzyme preparation. The mean values of three replicates and standard deviations are presented

The *S. commune* enzyme cocktail was then examined for the saccharification capacity on pretreated lignocellulosic substrates. After 24 h of hydrolysis at an enzyme loading of 20 mg protein/g glucan, a significantly higher amount of reducing sugars was released by the *S. commune* cocktail from all substrates tested compared to the commercial enzymes (Fig. 4). In particular, reducing sugar yield produced by the *S. commune* cocktail from partially delignified Jerusalem artichoke stalk was approximately fivefold as high as that released by the *T. longibrachiatum* preparation. For the *S. commune* enzyme cocktail, switchgrass was the most digestible of all four substrates in the enzymatic hydrolysis, followed by Jerusalem artichoke stalk, Miscanthus, and corn stover, respectively. The observed difference in the hydrolysis yields of pretreated lignocellulosic materials could be related to differing effects of the sodium chlorite treatment on the chemical composition of the resulting biomass residue (see Additional file 2: Table S1).

**Fig. 5:**
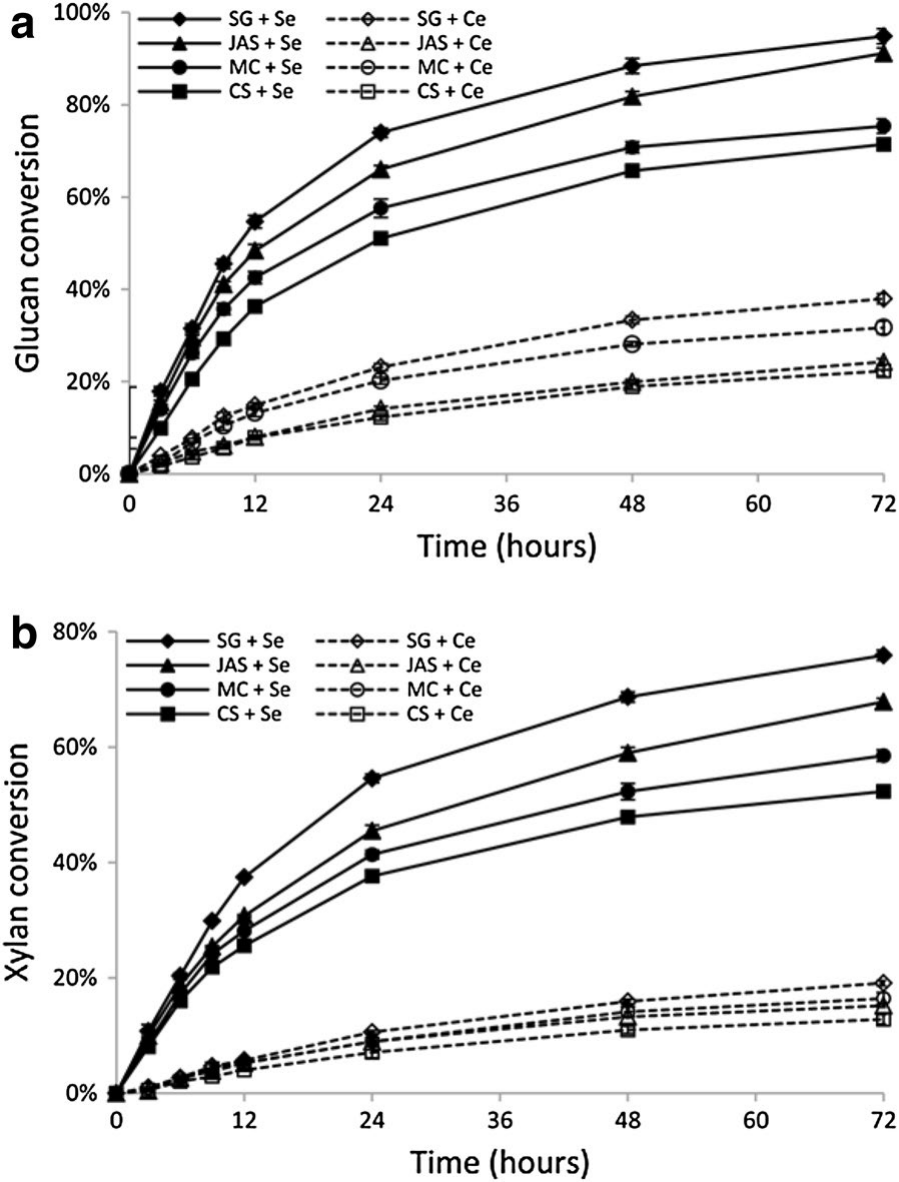
Conversion of (**a**) glucan and (**b**) xylan during hydrolysis of pretreated lignocellulosic substrates. The hydrolysis reactions were performed at 37 °C for 72 h incubation with 2 % (w/v) pretreated biomass. *S. commune* enzyme cocktail and commercial enzyme preparation were both dosed at 5 mg enzyme/g glucan. *SG* switchgrass, *JAS* Jerusalem artichoke stalk, *MC* Miscanthus, *CS* corn stover. *Se* *S. commune* enzyme cocktail, *Ce* commercial enzyme preparation. Each test was conducted in duplicate

The saccharification performance of the *S. commune* cocktail was further investigated at a relatively low enzyme loading of 5 mg protein/g glucan. After 72 h of hydrolysis, the highest glucan conversion (about 95 %) was achieved by the *S. commune* enzymes from pretreated switchgrass, 2.5-fold as high as that (about 38 %) by the commercial *T. longibrachiatum* preparation (Fig. 5a). Such remarkable conversion difference was also observed when saccharification was performed with the other pretreated substrates. In fact, the hydrolysis extents of glucan by the *S. commune* enzymes within 9 h already exceeded those by the commercial enzymes after 72 h. While conducting these saccharification experiments, we also found that the *S. commune* cocktail could achieve much higher xylan hydrolysis yields from pretreated biomass in comparison with the commercial preparation (Fig. 5b). For example, after 72 h the xylan conversion in the pretreated switchgrass by the *S. commune* enzymes was 75.9 %, while the commercial *T. longibrachiatum* preparation hydrolyzed only 19.1 % of the xylan in the same substrate. Time course of saccharification demonstrated that the enzyme cocktail prepared from *S. commune* was capable of higher levels of overall hydrolysis of pretreated biomass at a faster rate than the *T. longibrachiatum* preparation.

### Extracellular proteomic analysis

**Fig. 6:**
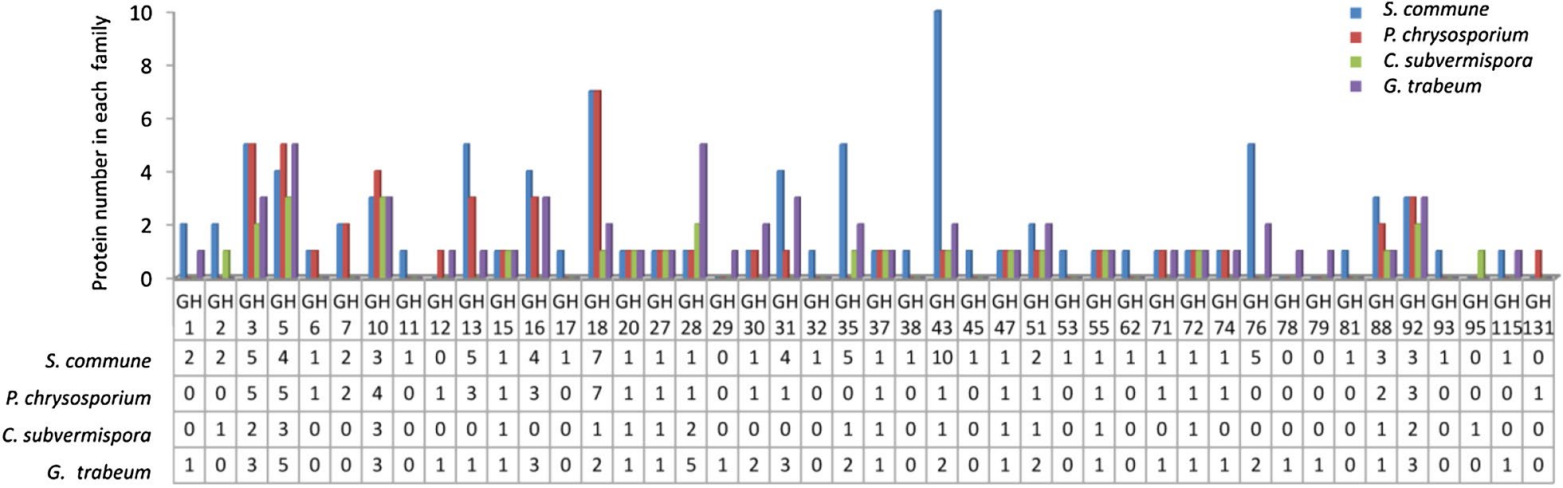
Distribution of glycosyl hydrolase (GH) family proteins identified in the secretomes of four fungi. GH family annotations of the secreted proteins are based on the carbohydrate-active enzyme database (CAZy)

Proteomic analysis using nanoLC-MS/MS was performed to compare the lignocellulolytic enzyme profile of the *S. commune* secretome with those of *P. chrysosporium*, *C. subvermispora* and *G. trabeum* during solid-state fermentation on Jerusalem artichoke stalk. A total of 229 proteins were identified in the *S. commune* secretome, 112 proteins were identified in the *P. chrysosporium* secretome, 95 proteins for *C. subvermispora*, and 109 for *G. trabeum* (see Additional file 3: Table S2). Four fungal species were noticeably different in the number of proteins from glycoside hydrolase (GH) and family distribution (Fig. 6). Enzymes produced by the four fungi can be classified into 38, 26, 19, and 30 GH families, respectively. Proteins from some GH families, such as GH3 (β-glucosidase), GH5 (endoglucanase), GH10 (endoxylanase), GH15 (glucoamylase), GH18 (chitinase), GH20 (N-acetylhexosaminidase), GH27 (α-galactosidase), GH28 (polygalacturonase), GH47 (1,2-α-mannosidase), GH51 (α-l-arabinofuranosidase), GH55 (exo-β-1,3-glucanase), GH88 (d-4,5-unsaturated glucuronyl hydrolase), and GH92 (α-mannosidase) were represented in all four species. A number of other GH families, including GH11 (endo-1,4-β-xylanase), GH17 (glucan endo-1,3-β-glucosidase), GH32 (arabinosidase), GH38 (1,2-α-mannosidase), GH45 (β-1,4-glucanase), GH53 (endo-β-1,4-galactanase), GH62 (α-l-arabinofuranosidase), GH81 (β-1,3-glucanase), and GH93 (exo-1,5-α-l-arabinanase), were detected exclusively in the secretome of *S. commune*, while hydrolases from GH29 (α-l-fucosidase), GH78 (α-l-rhamnosidase), and GH79 (β-glucuronidase) were represented in the *G. trabeum* secretome only. GH131 (exo-β-1,3/1,6-glucanase) was detected only in the *P. chrysosporium* secretome and GH95 (α-l-fucosidase) only in *C. subvermispora*. The classification results based on GH families revealed that *S. commune* produced a much larger battery of GH family representatives than those of the other three species.

**Fig. 7:**
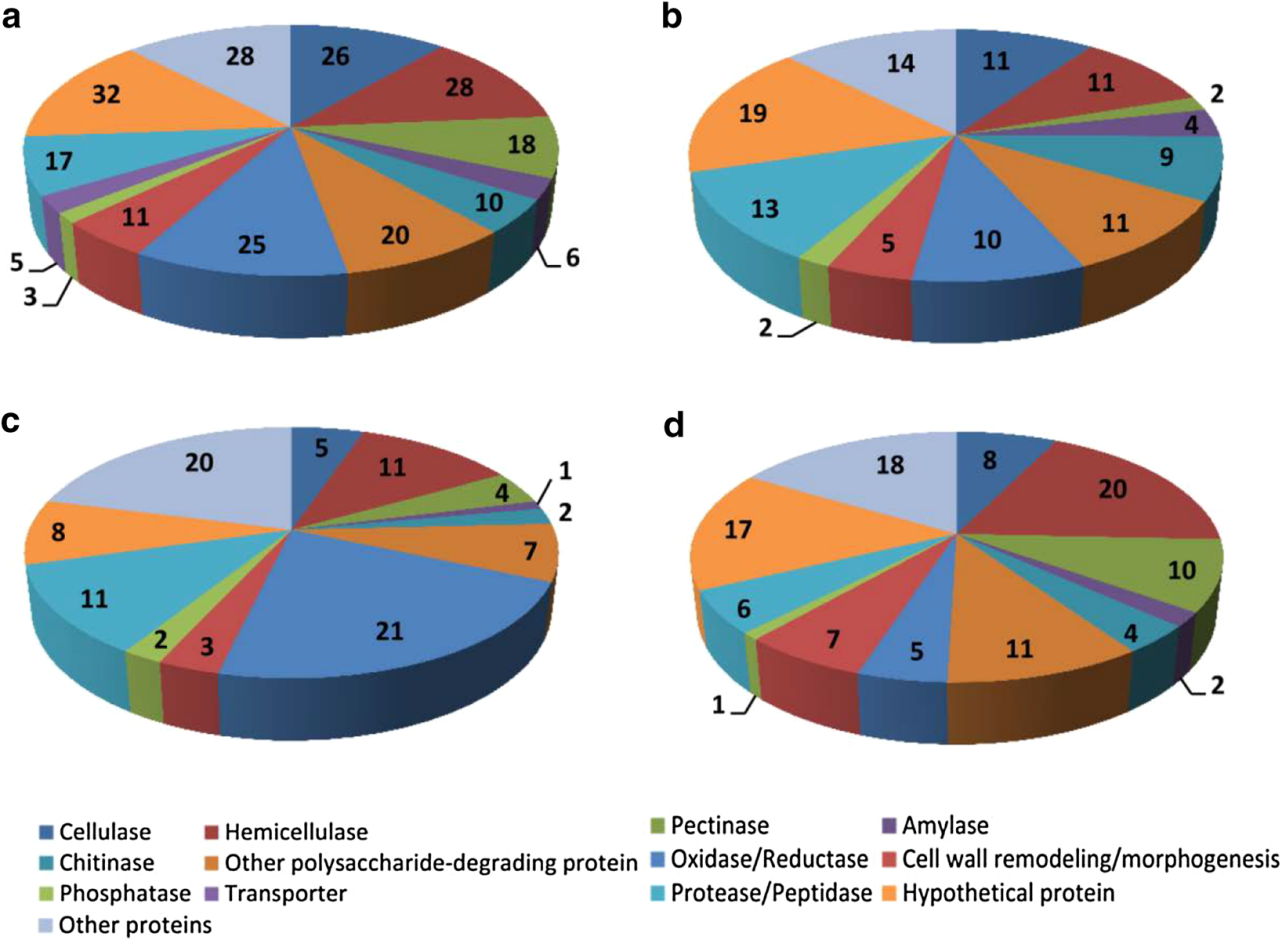
Functional classification of the proteins released by four fungi grown on Jerusalem artichoke stalk. Functional predictions of (**a**) *S. commune* proteins, (**b**) *P. chrysosporium* proteins, (**c**) *C. subvermispora* proteins, and (**d**) *G. trabeum* proteins are based on the Joint Genome Institute (JGI) databases for *Schizophyllum commune* H4-8 v3.0, *Phanerochaete chrysosporium* RP-78 v2.2, *Ceriporiopsis* (*Gelatoporia*) *subvermispora* B, and *Gloeophyllum trabeum* v1.0, respectively

Functional annotation analysis of the secreted proteins indicated that among the four fungal species, *S. commune* released the highest amount of extracellular enzymes involved in the degradation of all carbohydrate components of plant cell walls, including cellulose, hemicellulose, pectin, starch, and other polysaccharides (Fig. 7), which indicated its high capacity for enzyme secretion. The complete hydrolysis of cellulose depends on the complementary interaction of three different classes of core cellulases: endo-1,4-β-glucanases, exo-1,4-β-glucanases, and β-glucosidases [38, 39]. Both *P. chrysosporium* and *S. commune* produced a full enzymatic suite necessary for cellulose degradation (Table 4). Three endoglucanases (GH5), three exoglucanases (GH6 and GH7), and six β-glucosidases (GH1 and GH3) were detected in the *S. commune* secretome, and two endoglucanases (GH5), three exoglucanases (GH6 and GH7), and three β-glucosidases (GH3) were detected in *P. chrysosporium*. In contrast, identified cellulolytic enzymes of *C. subvermispora* comprised only two endoglucanases and two β-glucosidases and no exo-acting cellobiohydrolase was detected. As for *G. trabeum*, no GH6 or GH7 cellobiohydrolases were detected although its secretome contained three endoglucanases (GH5 and GH12) and four β-glucosidases (GH3 and GH30).

**Table 4:** Summary of cellulose-degrading proteins identified in the secretomes of four fungi

Species	Accession no.^a^	Family^b^	Predicted protein^a^	Up^c^	SignalP^d^
*S. commune*	Endoglucanase				
	Schco3|2602020	GH5	endo-β-1,4-glucanase	18	N
	Schco3|2151684	GH5	endo-β-1,4-glucanase	7	Y
	Schco3|17204	GH45	endo-β-1,4-glucanase	5	Y
	Exoglucanase				
	Schco3|17408	GH6	cellobiohydrolase	11	Y
	Schco3|13879	GH7	reducing end-acting cellobiohydrolase	17	Y
	Schco3|2361770	GH7	reducing end-acting cellobiohydrolase	12	Y
	Β-glucosidase				
	Schco3|2450343	GH1	β-glucosidase	9	N
	Schco3|105341	GH1	β-glucosidase	2	Y
	Schco3|2266284	GH3	β-glucosidase	16	N
	Schco3|2003814	GH3	β-glucosidase	13	Y
	Schco3|1099292	GH3	β-glucosidase	4	N
	Schco3|2026622	GH3	β-glucosidase	20	N
*P. chrysosporium*	Endoglucanase				
	Phchr2|2536644	GH5,CBM1	endoglucanase	2	Y
	Phchr2|2864492	GH5,CBM1	endoglucanase	2	Y
	Exoglucanase				
	Phchr2|133052	GH6,CBM1	exocellobiohydrolase	5	Y
	Phchr2|129072	GH7,CBM1	1,4-β-cellobiohydrolase	8	Y
	Phchr2|137372	GH7,CBM1	cellobiohydrolase	5	Y
	Β-glucosidase				
	Phchr2|139063	GH3	β-glucosidase	3	Y
	Phchr2|1854790	GH3	β-glucosidase	3	N
	Phchr2|2894234	GH3,CBM1	β-glucosidase	2	Y
*C. subvermispora*	Endoglucanase				
	Cersu1|106487	GH5	endoglucanase	7	N
	Cersu1|102329	GH5,CBM1	endoglucanase	2	Y
	Β-glucosidase				
	Cersu1|108602	GH3	β-glucosidase	13	Y
	Cersu1|103439	GH3	β-glucosidase	8	Y
*G. trabeum*	Endoglucanase				
	Glotr1_1|57704	GH5	endoglucanase	9	Y
	Glotr1_1|63180	GH5	endoglucanase	7	Y
	Glotr1_1|138821	GH12	endo-1,4-β-glucanase	4	Y
	Β-glucosidase				
	Glotr1_1|71534	GH3	β-glucosidase	14	Y
	Glotr1_1|75899	GH3	β-glucosidase	18	N
	Glotr1_1|46629	GH30	β-glucosidase	5	Y
	Glotr1_1|75778	GH30	β-glucosidase	7	Y

^a^Accession numbers and predicted proteins were obtained from the corresponding Joint Genome Institute (JGI) databases

^b^Family information was obtained from the carbohydrate-active enzyme database (CAZy)

^c^Unique peptides

^d^Prediction of signal peptides was based on SignalP analysis

Heteroxylan has a backbone of β-(1,4)-linked xylopyranosyl units that can be substituted with side groups. Heteroxylan degradation requires the concerted action of both main chain and side-group hemicellulases. All four fungi in this study produced a variety of enzymes involved in the xylan degradation, but differed in both the number and family distribution (Table 5). Present in all four secretomes were endo-1,4-β-xylanase from GH10, α-galactosidase from GH27, α-l-arabinofuranosidase from GH51, and acetyl xylan esterase from carbohydrate esterase (CE) family 1. In addition, *P. chrysosporium* secreted two endo-1,4-β-mannanases (GH5), one galactan 1,3-β-galactosidase (GH43), and one oligoxyloglucan cellobiohydrolase (GH74). *C. subvermispora* secretome contained two beta-mannosidases (GH2) and one beta-galactosidase (GH35) besides an α-l-fucosidase (GH95). Compared with the white-rot species, *S. commune* and *G. trabeum* expressed more hemicellulases cleaving xylan side chains (20 and 13, respectively). Specifically, side-chain-degrading hemicellulases of *S. commune* included three acetyl xylan esterases (CE1), one feruloyl esterase (CE1), one glucuronoyl esterase (CE15), one α-xylosidase (GH31), five β-galactosidases (GH35), six α-l-arabinofuranosidases (GH43, GH51 and GH62), and one α-glucuronidase (GH115).

**Table 5:** Summary of hemicellulose-degrading proteins identified in the secretomes of four fungi

Species	Accession no.^a^	Family^b^	Predicted protein^a^	Up^c^	SignalP^d^
*S. commune*	Main chain				
	Schco3|2357273	GH2	β-mannosidase	2	Y
	Schco3|2174600	GH2	β-mannosidase	18	Y
	Schco3|2573073	GH10	β-1,4-xylanase	4	N
	Schco3|2252725	GH10	β-1,4-xylanase	2	Y
	Schco3|2599608	GH10, CBM1	β-1,4-xylanase	12	Y
	Schco3|2170032	GH11	endo-1,4-β-xylanase A	7	Y
	Schco3|103086	GH43	β-xylosidase	2	Y
	Schco3|2147860	GH74	xyloglucanase	5	Y
	Side group				
	Schco3|2159785	GH27	α-galactosidase	7	N
	Schco3|2310213	GH31	α-xylosidase	1	Y
	Schco3|2304995	GH35	β-galactosidase	15	Y
	Schco3|2669535	GH35	β-galactosidase	1	Y
	Schco3|110618	GH35	β-galactosidase	1	Y
	Schco3|2138893	GH35	β-galactosidase	13	Y
	Schco3|13761	GH35	β-galactosidase	5	N
	Schco3|2347041	GH43	α-l-arabinofuranosidase	9	Y
	Schco3|15661	GH43	α-l-arabinofuranosidase	4	N
	Schco3|2226774	GH43	α-l-arabinofuranosidase	3	N
	Schco3|2085606	GH51	α-l-arabinofuranosidase	3	N
	Schco3|108819	GH51, CBM4	α-l-arabinofuranosidase	4	Y
	Schco3|2167580	GH62	α-l-arabinofuranosidase	5	Y
	Schco3|1339154	GH115	α-glucuronidase	8	Y
	Schco3|2275054	CE1	acetyl xylan esterase	3	Y
	Schco3|2061511	CE1, CBM1	acetyl xylan esterase	2	N
	Schco3|2061509	CE1, CBM1	acetyl xylan esterase	2	Y
	Schco3|1161522	CE1, CBM17	feruloyl esterase	4	Y
	Schco3|2151545	CE1	esterase	2	N
	Schco3|2138510	CE15	glucuronoyl esterase	6	Y
*P. chrysosporium*	Main chain				
	Phchr2|1969118	GH3	β-xylosidase	5	Y
	Phchr2|2686185	GH5, CBM1	endo-1,4-β-mannanase	2	Y
	Phchr2|2972357	GH5, CBM1	endo-1,4-β-mannanase	2	Y
	Phchr2|2970250	GH10	endo-β-1,4-xylanase	6	Y
	Phchr2|138715	GH10, CBM1	endo-1,4-β-xylanase C	1	Y
	Phchr2|2970992	GH10, CBM1	endo-1,4-β-xylanase	4	Y
	Phchr2|134556	GH74, CBM1	oligoxyloglucan cellobiohydrolase	8	Y
	Side group				
	Phchr2|125033	GH27	α-galactosidase	5	Y
	Phchr2|2973689	GH43, CBM35	galactan 1,3-β-galactosidase	2	Y
	Phchr2|2305691	GH51, CBM4	α-l-arabinofuranosidase	4	N
	Phchr2|126075	CE1, CBM1	acetyl xylan esterase	1	Y
*C. subvermispora*	Main chain				
	Cersu1|107994	GH2	β-mannosidase	5	Y
	Cersu1|103229	GH2	β-mannosidase	2	N
	Cersu1|157432	GH10	endo-1,4-β-xylanase	5	Y
	Cersu1|116326	GH10	endo-1,4-β-xylanase A	4	Y
	Cersu1|67561	GH10, CBM1	endo-1,4-β-xylanase A	3	Y
	Side group				
	Cersu1|102094	GH27	α-galactosidase	4	N
	Cersu1|124631	GH27	α-galactosidase	2	Y
	Cersu1|110070	GH35	β-galactosidase	18	Y
	Cersu1|99896	GH51	α-l-arabinofuranosidase A	4	Y
	Cersu1|106199	GH95	α-l-fucosidase	3	Y
	Cersu1|106142	CE1, CBM1	acetyl xylan esterase	2	Y
*G. trabeum*	Main chain				
	Glotr1_1|115191	GH2	β-mannosidase	13	Y
	Glotr1_1|122002	GH3	β-xylosidase	16	Y
	Glotr1_1|114574	GH5	endo-β-mannanase	10	Y
	Glotr1_1|46499	GH10	endo-1,4-β-xylanase	19	N
	Glotr1_1|138785	GH10	β-1,4-xylanase	5	Y
	Glotr1_1|140289	GH10, CBM1	endo-1,4-β-xylanase	12	Y
	Glotr1_1|68887	GH74	oligoxyloglucan cellobiohydrolase	21	Y
	Side group				
	Glotr1_1|81012	GH1	β-galactosidase-like protein	23	Y
	Glotr1_1|117566	GH27	α-galactosidase	12	Y
	Glotr1_1|61165	GH29	α-l-fucosidase	10	N
	Glotr1_1|141329	GH31	α-xylosidase	29	Y
	Glotr1_1|119185	GH31	α-xylosidase	19	Y
	Glotr1_1|81512	GH35	β-galactosidase	24	N
	Glotr1_1|111095	GH35	β-galactosidase	37	Y
	Glotr1_1|69366	GH43, CBM6	galactan 1,3-β-galactosidase	10	Y
	Glotr1_1|134804	GH51	α-l-arabinofuranosidase	14	Y
	Glotr1_1|111463	GH51, CBM4	α-l-arabinofuranosidase	12	Y
	Glotr1_1|121308	GH115	α-glucuronidase	24	Y
	Glotr1_1|117128	CE1	acetyl xylan esterase	10	Y
	Glotr1_1|46545	CE15	glucuronoyl esterase	7	Y

^a^ Accession numbers and predicted proteins were obtained from the corresponding Joint Genome Institute (JGI) databases

^b^ Family information was obtained from the carbohydrate-active enzyme database (CAZy)

^c^ Unique peptides

^d^ Prediction of signal peptides was based on SignalP analysis

Pectin is a highly branched structural heteropolysaccharide contained in the primary cell walls of terrestrial plants [40]. Because of the complexity of its structural and chemical composition, a broad range of enzymes are required for the degradation of pectin. Polygalacturonases from GH28 and pectin methylesterases from CE8 were represented in all four species (Table 6). *G. trabeum* produced the most endo- and exo-acting polygalacturonases and it also secreted an endo-1,5-α-l-arabinanase (GH43), an α-l-rhamnosidase (GH78), and a β-glucuronidase (GH79). As for *S. commune*, it produced a higher diversity of pectinolytic enzymes targeting side chains, including one rhamnogalacturonan lyase (PL4), four pectate lyases (PL1 and PL3), one endo-β-1,4-galactanase (GH53), one exo-1,5-α-l-arabinanase (GH93), and one rhamnogalacturonan acetylesterase (CE12).

**Table 6:** Summary of pectin-degrading proteins identified in the secretomes of four fungi

Species	Accession no.^a^	Family^b^	Predicted protein^a^	Up^c^	SignalP^d^
*S. commune*	Main chain				
	Schco3|110819	GH28	polygalacturonase	3	Y
	Schco3|2141311	PL1	pectate lyase	9	Y
	Schco3|2264630	PL1	pectate lyase	9	Y
	Schco3|2164161	PL3	pectate lyase	3	Y
	Schco3|2130270	PL3	pectate lyase	3	Y
	Schco3|2063343	PL4	rhamnogalacturonan lyase	12	Y
	Side group				
	Schco3|2287708	GH16	endo-β-1,3-galactanase	4	Y
	Schco3|2601007	GH16	endo-β-1,3-galactanase	4	Y
	Schco3|2189132	GH43	endo-1,5-α-l-arabinosidase	5	Y
	Schco3|2011638	GH43	α-l-arabinosidase	7	Y
	Schco3|2601132	GH43	α-l-arabinosidase	6	Y
	Schco3|2187351	GH43	exo-β-1,3-galactanase	1	Y
	Schco3|2086209	GH43	arabinosidase	3	Y
	Schco3|13837	GH43,CBM35	exo-β-1,3-galactanase	9	Y
	Schco3|15841	GH53	endo-β-1,4-galactanase	3	Y
	Schco3|15206	GH93	exo-1,5-α-l-arabinanase	4	Y
	Schco3|2183364	CE8	pectinesterase	7	N
	Schco3|231570	CE12	rhamnogalacturonan acetylesterase	6	Y
*P. chrysosporium*	Main chain				
	Phchr2|2865709	GH28	endo-polygalacturonase	7	Y
	Side group				
	Phchr2|132137	CE8	pectin methylesterase	3	Y
*C. subvermispora*	Main chain				
	Cersu1|105970	GH28	endo-polygalacturonase	7	Y
	Cersu1|124872	GH28	exo-polygalacturonase	2	Y
	Side group				
	Cersu1|112103	GH43	endo-1,5-α-l-arabinase	3	Y
	Cersu1|103663	CE8	pectin methylesterase	4	Y
*G. trabeum*	Main chain				
	Glotr1_1|6650	GH28	exo-polygalacturonase	7	N
	Glotr1_1|110574	GH28	endo-polygalacturonase	4	Y
	Glotr1_1|117232	GH28	exo-polygalacturonase	7	Y
	Glotr1_1|120615	GH28	endo-polygalacturonase	7	Y
	Glotr1_1|138836	GH28	exo-polygalacturonase	7	Y
	Side group				
	Glotr1_1|58475	GH43	endo-1,5-α-l-arabinanase	4	Y
	Glotr1_1|136552	GH78	α-l-rhamnosidase	12	Y
	Glotr1_1|116837	GH79	β-glucuronidase	10	N
	Glotr1_1|77537	CE8	pectin methylesterase	3	Y
	Glotr1_1|112531	CE8	pectin methylesterase	8	Y

^a^ Accession numbers and predicted proteins were obtained from the corresponding Joint Genome Institute (JGI) databases

^b^ Family information was obtained from the carbohydrate-active enzyme database (CAZy)

^c^ Unique peptides

^d^ Prediction of signal peptides was based on SignalP analysis

A clear difference between four fungi was observed in the number of non-hydrolytic proteins involved in polysaccharide depolymerization. Eight and three members of AA9 family were identified in the *S. commune* and *P. chrysosporium* secretomes, respectively, while only one was detected in *G. trabeum* (Table 7). No AA9 proteins were found in the *C. subvermispora* secretome. The *S. commune* secretome also contained three expansin-like proteins, as opposed to no such proteins found in the secretomes of the other three species.

**Table 7:** Summary of non-hydrolytic proteins involved in polysaccharide deconstruction identified in the secretomes of four fungi

Species	Accession no.^a^	Family^b^	Predicted protein^a^	Up^c^	SignalP^d^
*S. commune*	Schco3|1244931	AA9	lytic polysaccharide monooxygenase	4	Y
	Schco3|1324169	AA9	lytic polysaccharide monooxygenase	7	Y
	Schco3|1280218	AA9	lytic polysaccharide monooxygenase	3	Y
	Schco3|2601330	AA9	lytic polysaccharide monooxygenase	3	Y
	Schco3|1219608	AA9	lytic polysaccharide monooxygenase	3	Y
	Schco3|1105422	AA9	lytic polysaccharide monooxygenase	4	Y
	Schco3|1144769	AA9	lytic polysaccharide monooxygenase	3	Y
	Schco3|1192808	AA9	lytic polysaccharide monooxygenase	3	Y
	Schco3|2146679	CBM1	carbohydrate-binding module family 1 protein	11	Y
	Schco3|2246934	CBM13	carbohydrate-binding module family 13 protein	2	Y
	Schco3|2034642	CBM13	carbohydrate-binding module family 13 protein	2	Y
	Schco3|2195917	CBM63	expansin-like protein	3	Y
	Schco3|2195885	CBM63	expansin-like protein	5	Y
	Schco3|2437508	CBM63	expansin-like protein	2	Y
*P. chrysosporium*	Phchr2|2609667	AA9	lytic polysaccharide monooxygenase	3	Y
	Phchr2|122129	AA9	lytic polysaccharide monooxygenase	2	Y
	Phchr2|1841316	AA9	lytic polysaccharide monooxygenase	3	Y
*C. subvermispora*	Cersu1|100632	CBM13	carbohydrate-binding module family 13 protein	4	N
*G. trabeum*	Glotr1_1|63531	AA9	lytic polysaccharide monooxygenase	7	Y

^a^ Accession numbers and predicted proteins were obtained from the corresponding Joint Genome Institute (JGI) databases

^b^ Family information was obtained from the carbohydrate-active enzyme database (CAZy)

^c^ Unique peptides

^d^ Prediction of signal peptides was based on SignalP analysis

With regard to lignin degradation, the secretion patterns of oxidoreductases differed substantially between the four fungi (Table 8). Extracellular protein profile produced by *P. chrysosporium* included three lignin peroxidases and one manganese peroxidases, while two manganese peroxidases and one Lac were detected in the *C. subvermispora* secretome. In contrast, neither of the *S. commune* and *G. trabeum* secretomes contained ligninolytic enzymes such as manganese peroxidases, lignin peroxidases, or laccases. All fungal species secreted enzymes involved in peroxide generation, but were different in the family distribution and amount of members.

**Table 8:** Summary of oxidoreductases involved in lignin degradation identified in the secretomes of four fungi

Species	Accession no.^a^	Family^b^	Predicted protein^a^	Up^c^	SignalP^d^
*S. commune*	Peroxide generation				
	Schco3|2106675	AA3	aryl-alcohol oxidase	11	Y
	Schco3|2186806	AA3	glucose oxidase	14	Y
	Schco3|13888	AA3	glucose oxidase	14	Y
	Schco3|2443737	AA3	alcohol oxidase	9	N
	Schco3|2110714	AA3	alcohol oxidase	3	Y
	Schco3|1099997	AA3	alcohol oxidase	4	N
	Schco3|1110525	AA5	copper radical oxidase	2	Y
	Schco3|1133515	AA7	glucooligosaccharide oxidase	9	Y
	Schco3|1149633	AA7	glucooligosaccharide oxidase	2	Y
	Iron reduction				
	Schco3|114791	AA3	cellobiose dehydrogenase	14	Y
	Schco3|13527	AA6	1,4-benzoquinone reductase	5	Y
	Schco3|2599696		ferric reductase	4	Y
*P. chrysosporium*	Ligninolysis				
	Phchr2|1719525	AA2	Ligninase B	4	Y
	Phchr2|122202	AA2	Ligninase LG6	3	Y
	Phchr2|1385954	AA2	Ligninase precursor	2	Y
	Phchr2|1179466	AA2	Manganese peroxidase H3	5	Y
	Peroxide generation				
	Phchr2|11068	AA5	glyoxal oxidase precursor	4	Y
	Phchr2|134241	AA5	copper radical oxidase variant A	3	Y
	Phchr2|124009	AA5	copper radical oxidase	8	Y
	Iron reduction				
	Phchr2|11098	AA3	Cellobiose dehydrogenase	12	Y
*C. subvermispora*	Ligninolysis				
	Cersu1|108863	AA1	Laccase	3	Y
	Cersu1|107141	AA2	manganese-dependent peroxidase	7	Y
	Cersu1|22243	AA2	manganase peroxidase	3	Y
	Peroxide generation				
	Cersu1|107672	AA3	aryl-alcohol oxidase precursor	6	Y
	Cersu1|121682	AA3	alcohol oxidase	5	N
	Cersu1|106232	AA5	copper radical oxidase	1	Y
	Cersu1|106640	AA5	copper radical oxidase	3	Y
	Iron reduction				
	Cersu1|69058	AA6	1,4-benzoquinone reductase	5	N
*G. trabeum*	Peroxide generation				
	Glotr1_1|81501	AA3	aryl-alcohol oxidase	13	Y
	Glotr1_1|82487	AA3	aryl-alcohol oxidase-like protein	13	Y
	Glotr1_1|74773	AA3	alcohol oxidase	11	Y
	Glotr1_1|65654	AA5	glyoxal oxidase	8	Y
	Iron reduction				
	Glotr1_1|113732	AA3	cellobiose dehydrogenase	3	N

^a^ Accession numbers and predicted proteins were obtained from the corresponding Joint Genome Institute (JGI) databases

^b^ Family information was obtained from the carbohydrate-active enzyme database (CAZy)

^c^ Unique peptides

^d^ Prediction of signal peptides was based on SignalP analysis

## Discussion

During SEM and stalk component degradation analysis, it is interesting to note that *S. commune*, with the highest degradative enzyme activities after 15 days, did not produce strong polysaccharide degradation in Jerusalem artichoke stalk. *P. chrysosporium*, on the other hand, showed the highest consumption of cellulose and hemicellulose despite moderate enzyme activities. The inconsistency in the enzyme activities and degradation kinetics can be explained by the different lignin-degrading strategies employed by different fungi. In plant cell walls, lignin is intimately associated with structural polysaccharides. The complex hydrophobic network of lignin acts as a form of protection for the interior carbohydrate components, thus making them recalcitrant to hydrolytic attack [41]. Although they cannot utilize lignin as a carbon source, both *P. chrysosporium* and C*. subvermispora* secreted a wide array of extracellular peroxidases and oxidases to efficiently depolymerize and mineralize this formidable substrate. The interaction of ligninolytic enzymes resulted in the degradation of lignin and consequently, the exposure of the polysaccharide constituents of plant cell walls, rendering them more available to classical cellulases and hemicellulases. In contrast, the brown-rot fungus *G. trabeum* has been suggested to employ hydroquinone-driven Fenton chemistry as a degradative mechanism for initiating polysaccharide depolymerization [23]. Similar to *G. trabeum*, the *S. commune* secretome did not contain the core components of ligninolytic enzyme system. It caused a very limited lignin removal from the untreated stalk due to the lack of extracellular class II peroxidases like MnPs and LiPs. The huge bulk of lignin not only impedes enzymatic access to cell wall polysaccharides but also causes non-productive adsorption of hydrolytic enzymes [42]. Despite the incomplete ligninolysis, *S. commune* still caused an appreciable degradation of polysaccharides in Jerusalem artichoke stalk compared with *G. trabeum*.

During the lignocellulose biodegradation *S. commune* demonstrated a preferential degradation of polysaccharides with respect to lignin. The pyrolysis analysis provided evidence that attack of plant cell wall constituents by *S. commune* also resulted in some modifications of lignin substructure, including demethoxylation, demethylation, and side-chain oxidation. Such oxidative alterations of lignin moieties were very similar to that by the brown-rot fungus *G. trabeum*. Although the lignin residues after hydroxyl radical attack remain in situ, the original inter-monomer side-chain linkages within lignin have been disrupted and the resulting aromatic polymer is no longer recognizable as lignin [43]. It is probable that *S. commune* utilized an oxidative mechanism to enable the diffusion of carbohydrate-degrading enzymes through the lignocellulosic matrix to interior polysaccharides.

In the lignin degradation systems of white-rot fungi, an array of redox enzymes, such as copper radical oxidases and glucose-methanol-choline (GMC) oxidoreductases, are physiologically coupled to peroxidases via H_2_O_2_ generation. In view of Fenton chemistry employed by brown-rot fungi, H_2_O_2_ production by copper radical oxidases and GMC oxidoreductases potentially supports hydroxyl radical generation. In the absence of class II peroxidases, it is interesting to note that *S. commune* released a more diverse assortment of oxidoreductases than *P. chrysosporium* and *C. subvermispora*. Specifically, we observed a high number of GMC oxidoreductases in the protein profile of the *S. commune* secretome relative to the other two species, including an aryl-alcohol oxidase (>jgi|Schco3|2106675), two glucose oxidases (>jgi|Schco3|2186806 and >jgi|Schco3|13888), and three alcohol oxidase (>jgi|Schco3|2443737, >jgi|Schco3|2110714, and >jgi|Schco3|1099997). It has been found that alcohol oxidases could use methanol, the primary product of lignin demethylation by brown-rot fungi, as a substrate to generate H_2_O_2_ [44]. Likewise, copper radical oxidase (>jgi|Schco3|1110525) and glucooligosaccharide oxidase (>jgi|Schco3|1133515) played a part in extracellular peroxide generation. Given the decay pattern of *S. commune* observed during stalk degradation, there remains the possibility that these seemingly redundant oxidoreductases serve as a source of H_2_O_2_ to support Fenton chemistry.

This proposed non-enzymatic oxidative mechanism was further supported by the fact that S. *commune* secreted multiple reductive enzymes involved in the Fe^2+^ production required for Fenton chemistry. In this connection, cellobiose dehydrogenase (CDH) (>jgi|Schco3|114791) and ferric reductase (>jgi|Schco3|2599696) may be of relevance to Fenton chemistry by their role in the Fe^3+^-reduction [45, 46]. Additionally, benzoquinone reductase (>jgi|Schco3|13527) and phenylalanine ammonia lyase (>jgi|Schco3|1191717) may participate in iron reduction system driven by quinone redox cycling [46]. These observations indicated that *S. commune* possessed the enzymatic apparatus to carry out Fenton reactions during lignocellulose degradation. The identification of these proteins in the *S. commune* secretome, viewed together with the production of iron-reducing agents and oxidative modification of lignin structure, supported a biodegradative role for extracellular Fenton system in plant cell wall degradation by this fungus.

In the conversion process of lignocellulosic feedstocks to bioethanol, the complete hydrolysis of structural polysaccharides into fermentable sugars requires the cooperative action of various degradative enzymes with complementary activities. In this context, *S. commune* is a promising enzyme source as its enzyme system favored a more complex inventory of polysaccharide-degrading enzymes with enhanced enzyme activities. Comparison of the lignocellulolytic enzyme profiles of *S. commune* and other fungi in the study showed that *S. commune* had significantly higher levels of hydrolytic enzyme activities, including cellulolytic, xylanolytic, and pectinolytic activities. Enzyme cocktail from *S. commune* showed more than threefold higher xylanase activity and twofold higher polygalacturonase activity than the *T. longibrachiatum* enzyme preparation with comparable levels of cellulolytic activities. Proteomic analysis revealed that its secretome contained a more diverse repertoire of GH families and more members in each family than the other fungi. Biological function analysis of extracellular proteins indicated that *S. commune* produced a larger variety of accessory enzymes that facilitate the complete hydrolysis of non-cellulosic polysaccharides, such as pectin and heteroxylan.

The present study showed that *S. commune* secreted a wide variety of hemicellulases and pectinases acting not only on main chains (xylanase, polygalacturonase, and pectate lyase), but also side groups, such as acetyl xylan esterase, α-l-arabinofuranosidase, α-glucuronidase, feruloyl esterase, endo-β-1,3-galactanase, and α-l-arabinosidase. It is noteworthy that the complementary action of cellulases and accessory enzymes such as hemicellulases, pectinases, and LPMOs, plays a key role in the hydrolysis of lignocellulosic materials as observed in previous studies. Xylanase supplementation to cellulase enzyme mixtures has been shown to improve the overall hydrolysis of pretreated lignocellulosic substrates due to increased cellulose accessibility to cellulases through solubilization of xylan and alteration of fiber features [47]. A notable improvement in cellulose hydrolysis was also observed when a pectinase-enriched complex was added to cellulase preparations [4]. Side-chain-degrading enzymes may also contribute to the conversion yields of lignocellulosic materials containing branched polysaccharides by enhancing the hydrolysis effectiveness of main chain-cleaving enzymes. It has been reported that acetyl xylan esterases present in enzyme mixtures could promote xylan solubilization, and thus the subsequent hydrolysis of xylan and cellulose in the biomass by removing acetyl groups from xylan, as demonstrated in the saccharification of wheat straw and giant weed [48]. When a very low amount (1.2 mg/g glucan) of bacterial α-arabinofuranosidase (GH51) and α-glucuronidase (GH67) was added to a mixture of cellulase and xylanase, the total enzyme loading required for a 80 % sugar yield from AFEX-pretreated corn stover can be decreased by about 33 % [49]. As most commercial cellulase preparations contain relatively low levels of hemicellulases and pectinases, the enzyme pool of *S. commune* via solid-state fermentation offers a preferable alternative as it favors a complex cocktail of synergistically acting accessory enzymes, which promote enzyme loading reduction without decreasing hydrolysis yields.

Comparison with the other wood-rotting fungi showed that in addition to glycoside hydrolases, *S. commune* produced higher amounts of LPMOs, expansin-like proteins together with carbohydrate-binding modules. These non-hydrolytic proteins are suggested to reduce the recalcitrance of the lignocellulosic matrix, thereby enhancing the efficiency of enzymatic hydrolysis by hydrolases. AA9 family is a recently discovered class of fungal copper-dependent polysaccharide monooxygenases that oxidatively cleave cellulose [39]. By introducing chain breaks in the crystalline cellulose microfibrils, LPMOs from AA9 family enhance cellulosic digestibility by conventional hydrolytic enzymes. Intensive studies have shown that LPMO addition can significantly boost the hydrolysis efficiency of cellulase mixtures [50, 51]. Several fungal and bacterial expansins and expansin-like proteins have been reported to improve the enzymatic hydrolysis of crystalline cellulose by cellulases [52–54] and xylan by xylanases [55] without detectable hydrolytic activity. More recently, a novel expansin from *S. commune*, ScExlx1 has been found to act on both cellulose and chitin [56]. It is suggested that expansins are able to loosen the tightly packed architecture of plant cell wall matrix through the disruption of hydrogen bonds in polysaccharide networks. The high abundance of non-hydrolytic proteins such as LPMOs and expansin-like proteins in the *S. commune* secretome suggested that they may contribute to the high saccharification capacity of the *S. commune* enzyme cocktail observed in this work.

The commercial *T. longibrachiatum* preparation used for comparison in our study contained a variety of hydrolytic enzymes, including cellulase, xylanase, pectinase, mannanase, xyloglucanase, laminarase, β-glucosidase, β-xylosidase, α-l-arabinofuranosidase, and amylase. The enzyme preparation has been used in the hydrolysis of alkaline-pretreated *Miscanthus giganteus* and achieved higher hydrolysis yield of polysaccharides than another commonly used cellulase preparation Celluclast 1.5L [57]. During the saccharification experiments, the *S. commune* cocktail exhibited high activity compared to the *T. longibrachiatum* preparation against all substrates tested, converting 71–95 % of glucan compared to 22–38 % glucan conversion by the commercial preparation. It should be noted that all the comparative hydrolysis in this study were carried out at the optimal temperature and pH of the commercial preparation. One would expect that when the optimal conditions for the *S. commune* enzymes were used, the effectiveness of enzymatic hydrolysis by the *S. commune* cocktail can be further improved. Additionally, most commercial cellulase mixtures need the supplementation of exogenous β-glucosidase for efficient hydrolysis due to end-product inhibition. The β-glucosidase activity in the *S. commune* enzyme mixture was sufficiently high to ensure a complete hydrolysis of cellobiose to glucose.

When using LC-MS/MS to analyze the extracellular proteome, we noticed that the expression patterns of some homologous proteins differed among fungi. For instance, both *P. chrysosporium* and *S. commune* encode two glucuronoyl esterases (CE15) in their genomes [58, 59], and we identified one CE15 protein (Schco3|2138510) in the *S. commune* secretome. However, no peptides matching CE15 proteins of *P. chrysosporium* were detected in our study, even though an earlier study detected such protein in extracellular liquid cultures [60]. It should be noted that the LC-MS/MS approach reported here favored identification of soluble proteins, whereas proteins bound to the solid substrate may be overlooked. As shown by SEM, the hyphae of *P. chrysosporium* penetrated through the stalk cell walls, while the mycelia of *S. commune* mostly covered the stalk surface over the cultivation period. As the growth of *P. chrysosporium* on stalk was characterized by invasive mycelia, we assumed that some extracellular proteins were adsorbed on the substrate colonized by fungi. Consequently, protein recovery from such closely intertwined substrate/mycelia mixture might be restricted by the extraction method. Therefore, growth conditions should be taken into account when it comes to the interpretation of the absence of detectable extracellular proteins.

## Conclusions

The current work described for the first time the decay pattern and the composition of the secretome of *S. commune* growing on complex lignocellulosic substrates. Comparison with the representatives of white-rot and brown-rot fungi revealed that *S. commune* employed a Fenton chemistry-based oxidative mechanism for lignocellulose modification, while possessing a composite polysaccharide-degrading enzyme system. Furthermore, the enzymatic cocktail derived from *S. commune* displayed high hydrolysis effectiveness of polysaccharide in lignocellulose materials at low protein loadings. Proteomic analysis of the *S. commune* extracellular enzyme profile indicated that the significant saccharification efficiency could be attributed to the synergistic cooperation of cellulases and hemicellulases, pectinases, and non-hydrolytic proteins like LPMOs and expansins. These findings presented here shed light on the complex degradative enzyme system of *S. commune*, and highlight the important role of accessory enzymes in lignocellulose hydrolysis.

## Methods

### Fungal strains and culture conditions

Fungal strain *S. commune* SH12 was isolated from forest soil (Shaanxi province, China) and preserved on potato dextrose agar (PDA) plates at 4 °C. *P. chrysosporium* PC2 were obtained from China General Microbiological Culture Collection Center (CGMCC) and *C. subvermispora* CBS 347.63 were obtained from the fungal culture collections of the Centraalbureau voor Schimmelcultures (CBS, Baarn, The Netherlands). These strains were maintained following suppliers’ protocol at 4 °C.Prior to solid-state fermentation, the fungi were cultivated at 28 °C for 7 days on PDA-medium plates.

Jerusalem artichoke stalk, mainly composed of 38.64 % cellulose, 22.58 % arabinoxylan (consisted of 18.05 % xylose and 4.53 % arabinose), 4.97 % pectin and 18.10 % lignin, was chopped into small chips of approximately 1 cm squares. The solid-state fermentation was carried out in 150 mL Erlenmeyer flasks containing 3 g of sterilized Jerusalem artichoke stalk and 10 ml of Mandels’ salts solution (pH 6.5, 95 ml of water, 5 ml 20X nitrate salts, and 0.1 ml 1000X trace elements). Each flask was inoculated with four mycelium agar plugs of 1 cm in diameter from fungal species and incubated at 28 °C without humidity control. Non-inoculated Jerusalem artichoke stalk incubated under the same conditions was used as control. All treatments were performed in triplicate.

### Scanning electron microscopy (SEM)

Scanning electron microscopy (SEM) was carried out to observe the morphological changes on Jerusalem artichoke stalk after 30 days of incubation with respective fungal species. Samples were prepared for scanning electron microscopy as described by [61]. Micrographs of non-inoculated and fungi-inoculated samples were taken using a Hitachi S3400N scanning electron microscope (Hitachi) at an acceleration voltage of 15.0 kV.

### Pyrolysis gas chromatography–mass spectrometry

Pyrolysis gas chromatography–mass spectrometry (Py-GC/MS) of sound and degraded stalk was performed using a EGA/PY-3030D pyrolyzer (Frontier Laboratories, Japan) coupled to a Shimadzu single quadrupole GCMS-QP2010 Ultra gas chromatograph–mass spectrometer (Shimadzu Scientific, Japan) with a Phenomenex Zebron™ ZB-5HT GC capillary column (30 m × 0.25 mm × 0.25 μm). The pyrolysis was conducted at 500 °C. The chromatography temperature was programmed from 45 °C for 3 min to 300 °C at 15 °C min^−1^ and then held for 5 min. Helium was used as carrier gas at a rate of 1 ml min^−1^. The pyrolysis products were identified by comparing the mass spectra with those of the Wiley and NIST libraries.

### Extracellular protein extraction

The cultures of each fungus were harvested every 5 days until the end of incubation. The fungal cultures were incubated with 30 mL Milli-Q water at 4 °C with shaking at a rate of 200 rpm for 2 h. The water-soluble phases containing the total proteins were collected by centrifugation at 10,000× *g* for 15 min at 4 °C and then passed through a 0.25-μm filter. The filtrates were assayed directly for enzyme activities before further concentration and desalting with 3-kDa cutoff Amicon Ultra-15 Centrifugal Filter Unit (Merck Millipore) and lyophilized for secretome analysis. The fungal mycelia were removed from the solid residues by incubating in a mixture of acetic acid and nitric acid (8:1, v/v) on ice with agitation for 1 h, and washed with deionized water until it was pH-neutral. The mycelium-free residues were oven-dried at 60 °C to constant weight for the subsequent determination of chemical composition. The structural carbohydrate and lignin contents were determined according to the NREL laboratory analytical procedure (version 08-03-2012). In brief, 0.5 g of sample (dry weight) was extracted with 200 mL of ethanol at 95 °C and hydrolyzed at 30 °C with 3.0 mL H_2_SO_4_ (72 %) for 60 min. Then 84 mL of water was added and a second hydrolysis was carried out in the autoclave at 121 °C for 1 h. The mixture was then filtered by porcelain filter crucibles with glass filters. The amounts of sugars in the filtrates were determined by HPLC. The content of Klason lignin was determined by subtracting the ash content from the solid residue dried at 105 °C overnight. Ash content was determined by heating the solid residue at 575 °C for 3 h.

### Enzyme assays

Activities of endoglucanase, endoxylanase, and polygalacturonase were determined by the dinitrosalicylic acid (DNS) assay [62], with 1.0 % low-viscosity carboxymethylcellulose (CMC), beechwood xylan, and polygalacturonic acid as substrates, respectively. 50 μL crude enzyme was incubated with 150 μL of 1.0 % (w/v) substrate in 50 mM sodium acetate buffer (pH 5.0) at 50 °C for 10 min, and the reaction was stopped with 50 μL of 1M NaOH. Calculation of enzyme activities was based on corresponding standards containing glucose, xylose, and galacturonic acid. Enzyme activities are represented in units per gram of dry substrate (U/gds), with one unit of enzyme activity defined as the amount of enzyme needed to liberate 1 μmol of reducing sugars in 1 min from the substrate under given assay conditions.

Cellobiohydrolase, β-glucosidase, and β-xylosidase activities were quantified using the respective substrates p-nitrophenyl-β-d-cellobioside (pNPC), p-nitrophenyl-β-d-glucopyranoside (pNPG), and p-nitrophenyl-β-d-xylopyranoside (pNPX) [63]. The reactions were carried out in 1.5 ml centrifuge tubes containing 50 μL crude enzyme, 50 μL of 200 mM sodium acetate buffer (pH 5.0), and 100 μL of 5 mM substrate. After 10 min incubation at 50 °C, the reaction was terminated by adding 100 μL 1M Na_2_CO_3_, and the color development was recorded at 405 nm. Enzyme activities were calculated using p-nitrophenol standard. One unit of enzyme activity was defined as the amount of enzyme that produced 1 μmol of pNP in 1 min under the assay conditions.

MnP activity was measured by the oxidative dimerization of 2,6-dimethoxyphenol (2,6-DMP) in the presence of H_2_O_2_ [64]. The reaction mixture contained 1 mM 2,6-dimethoxyphenol (2,6-DMP), 1 mM MnSO_4_, 50 mM sodium tartrate (pH 4.5), 100 μM H_2_O_2_, and crude enzyme in a total volume of 1000 μL. LiP activity was assayed with veratryl alcohol as a substrate in 100 mM sodium tartrate (pH 3.0) [65]. The crude enzyme was mixed with 40 mM veratryl alcohol and 10 mM H_2_O_2_ in a total volume of 1000 μL at room temperature. Laccase (Lac) activity was determined by monitoring the oxidation of 2,2′-azonodi-3-ethylbenzothiazoline-6-sulfuric acid (ABTS) at room temperature [66]. The reaction mixture contained 1 mM ABTS in 100 mM sodium acetate buffer (pH 4.5) and crude enzyme in a total volume of 1000 μL. One unit of enzyme activity was defined as the amount of enzyme that catalyzed the formation of 1 μmol of corresponding products in one min under the assay conditions.

### Reducing capacity analysis

Assessment of the iron-reducing ability of extracellular soluble extracts from fungal species was based on the formation of the Fe^2+^-ferrozine complex [67]. The reaction mixture contained 200 μL of 20 mM sodium acetate buffer (pH 5.0), 60 μL of 40 mM ferrozine, 100 μL sample, and 40 μL of 10 mM freshly prepared FeCl_3_. The absorbance change at 562 nm was recorded after 10 min of incubation at room temperature.

### Biomass saccharification

All lignocellulosic substrates were delignified with sodium chlorite before enzymatic hydrolysis [68]. Enzyme preparations used in the saccharification assay were crude enzyme cocktail from *S. commune* and a cellulase cocktail from *Trichoderma longibrachiatum* (C9748, Sigma). The hydrolysis reactions were carried out with 2 % (w/v) pretreated biomass in 50 mM sodium acetate buffer (pH 5.0) in a final volume of 1 ml. Saccharifications were performed at two enzyme loadings (20 and 5 mg protein/g delignified biomass) in an orbital shaker incubator at 37 °C. Control assays including enzyme without substrate and substrate without enzyme are conducted under the same conditions. Protein concentration was determined using the Bradford Protein Assay Kit (GenStar, China) according to the manufacturer’s instructions.

The hydrolysis was terminated by heating the reaction mixture at 100 °C for 10 min to inactivate the enzymes. The supernatants were collected by centrifugation at 12,000 rpm for 10 min and further clarified by filtration through a 0.25-μm filter. The total contents of reducing sugars were quantified by the DNS method. The individual concentration of glucose, xylose, and arabinose in the supernatants was determined by Essentia LC-15C high performance liquid chromatography (Shimadzu, Japan) equipped with an Rezex ROA-Organic acid H+ (8 %) column (Phenomenex, USA) and a RID-10A refractive index detector (Shimadzu, Japan). The glucan and xylan conversions were calculated based on the initial contents of polysaccharides in the pretreated substrates. All hydrolysis experiments were conducted in duplicate.

### Protein identification by nanoLC-MS/MS

50 μg of total protein was separated by SDS-PAGE in triplicate using 12.5 % polyacrylamide at 100 V, stained with Coomassie Blue G-250 overnight. Each lane was divided into six fractions, and each fraction was cut into small pieces (approximately 1 mm^2^). The gel pieces were washed with pure water for 3 times, destained with 10 % acetic acid, and washed again with pure water. The gel pieces were immerged in acetonitrile and then vacuum-dried. The dried gel pieces were incubated in 10 mM dithiothreitol (DTT) solution at 56 °C for 45 min, after which they were alkylated with 55 mM iodoacetamide (IAM) solution in the dark at room temperature for 30 min. The gel pieces were washed with acetonitrile again before vacuum-dried. Then the gel pieces were subject to trypsin digestion at 37 °C overnight. The peptides were extracted with 30 % acetonitrile in 0.1 % formic acid for 30 min followed by 60 % acetonitrile for 30 min, and dried in vacuum concentrator. The residue was reconstituted with 0.1 % formic acid for nanoLC-MS/MS analysis.

NanoLC separation was achieved with a Waters (Milford, MA, USA) nanoACQUITY nano-HPLC. Both trap column and analytical column were home made. The trap column was made with 100 μm I.D. fused silica capillary (Polymicro, Phoenix, AZ, USA) filled with 2 cm of C18 stationary phase (Phenomenex, Torrance, CA, USA). The analytical column was made with 50 μm I.D. fused silica capillary (Polymicro) filled with 10 cm of C18 stationary phase. A 3-μm-diameter spray tip was pulled at the end of the analytical column with a Sutter P-2000 laser micropipette puller (Sutter Instrument, Novato, CA, USA). A gradient elution program was used. Gradients of different lengths were used for samples of different complexities. Following LC nanospray, ESI-MS was performed on a Thermo Q Exactive high-resolution mass spectrometer (Thermo Scientific, Waltham, MA, USA). The 10 most intensive peptide signals from the full scan were selected for MS/MS scans.

Raw data from the mass spectrometer were preprocessed with Mascot Distiller 2.5 for peak picking. The resulting peak lists were searched against the Joint Genome Institute (JGI) databases for *S. commune* H4-8 v3.0, *P. chrysosporium* RP-78 v2.2, and *C.* (*Gelatoporia*) *subvermispora* B using Mascot search engine (version 2.5.1, Matrix Science). The search parameters included carbamidomethyl cysteine as fixed modification and oxidized methionine as the variable modification. A maximum of two missed tryptic cleavages was allowed. The peptide mass tolerance was set at 15 ppm, and MS/MS fragment mass tolerance was set at 0.02 Da. For protein identification, only proteins containing a minimum of two significant peptides were considered and at least one unique peptide needed to be identified in the protein. The significance threshold p is set to be less than 0.01, thus bringing peptide and protein false discovery rate (FDR) below 1 %. For each identified protein, the theoretical isoelectric point (pI) and molecular weight (Mw) was calculated using the Compute pI/Mw tool in ExPASy (http://web.expasy.org/compute_pi). SignalP (http://www.cbs.dtu.dk/services/SignalP) was used to predict possible secretion signals in the identified proteins. A schematic representation of the nanoLC-MS/MS procedure is shown in Additional file 4: Figure S2.

## Additional files

10.1186/s13104-016-1932-7 Pectin lyase and lignin peroxidase activities of four fungi during SSF on Jerusalem artichoke stalk. Pectin lyase activities are shown in A and lignin peroxidase activities are shown in B. The values shown are the mean of three replicates and the error bars indicate standard deviations from the mean values.
10.1186/s13104-016-1932-7 Chemical composition of lignocellulosic substrates before and after sodium chlorite delignification. The main components of lignocellulosic substrates include anhydroglucose (Glu), anhydroxylose (Xyl) and anhydroarabinose (Ara). The data represent the mean values of three replicates.
10.1186/s13104-016-1932-7 Identified proteins in the secretomes of four fungi during SSF on Jerusalem artichoke stalk.
10.1186/s13104-016-1932-7 Schematic representation of nanoLC-MS/MS analysis of the extracellular proteins.

## Abbreviations

SSF: solid-state fermentation
LPMO: lytic polysaccharide monooxygenase
CAZy: carbohydrate-active enzyme database
AA: auxiliary activity
NanoLC-MS/MS: nano liquid chromatography-tandem mass spectrometry
Py-GC/MS: pyrolysis coupled with gas chromatography-mass spectrometry
MnP: manganese peroxidase
LiP: lignin peroxidase
Lac: laccase
GH: glycoside hydrolase
CE: carbohydrate esterase
PL: polysaccharide lyase
CDH: cellobiose dehydrogenase
GMC oxidoreductase: glucose-methanol-choline oxidoreductase
CMC: carboxymethylcellulose
2,6-DMP: 2,6-dimethoxyphenol
ABTS: 2,2′-azonodi-3-ethylbenzothiazoline-6-sulfuric acid
DNS: 3,5-Dinitrosalicylic acid
pNPC: p-nitrophenyl-β-d-cellobioside
pNPG: p-nitrophenyl-β-d-glucopyranoside
pNPX: p-nitrophenyl-β-d-xylopyranoside
DTT: dithiothreitol
IAM: iodoacetamide
FDR: false discovery rate
