# Supplementary material for: Comparative analysis of the secretomes of Schizophyllum commune and other wood-decay basidiomycetes during solid-state fermentation reveals its unique lignocellulose-degrading enzyme system
Source: Biotechnol Biofuels. 2016 Feb 20;9:42. doi: 10.1186/s13068-016-0461-x (PMC4761152; doi:10.1186/s13068-016-0461-x)
Supplement: Supplementary file 1 — 10.1186/s13104-016-1932-7 Pectin lyase and lignin peroxidase activities of four fungi during SSF on Jerusalem artichoke stalk. Pectin lyase activities are shown in A and lignin peroxidase activities are shown in B. The values shown are the mean of three replicates and the error bars indicate standard deviations from the mean values. [file 13068_2016_461_MOESM1_ESM.docx]

**B**

**A**
