# Supplementary material for: Comparative analysis of the secretomes of Schizophyllum commune and other wood-decay basidiomycetes during solid-state fermentation reveals its unique lignocellulose-degrading enzyme system
Source: Biotechnol Biofuels. 2016 Feb 20;9:42. doi: 10.1186/s13068-016-0461-x (PMC4761152; doi:10.1186/s13068-016-0461-x)
Supplement: Supplementary file 2 — 10.1186/s13104-016-1932-7 Chemical composition of lignocellulosic substrates before and after sodium chlorite delignification. The main components of lignocellulosic substrates include anhydroglucose (Glu), anhydroxylose (Xyl) and anhydroarabinose (Ara). The data represent the mean values of three replicates. [file 13068_2016_461_MOESM2_ESM.docx]

**Table S1 Chemical composition of lignocellulosic substrates before and after sodium chlorite delignification**

| Substrate | Composition of raw substrate | | | |  | Composition of delignified substrate | | | |
| --- | --- | --- | --- | --- | --- | --- | --- | --- | --- |
|  | **Glu** | **Xyl** | **Ara** | **Lignin** |  | **Glu** | **Xyl** | **Ara** | **Lignin** |
| Switchgrass | 43.10 | 21.88 | 2.55 | 25.90 |  | 50.27 | 27.52 | 3.93 | 2.41 |
| Jerusalem artichoke stalk | 38.64 | 18.05 | 4.53 | 18.10 |  | 46.48 | 26.70 | 6.12 | 1.41 |
| Miscanthus | 34.71 | 17.44 | 2.76 | 19.19 |  | 49.64 | 23.37 | 3.09 | 8.10 |
| Corn stover | 40.01 | 14.60 | 2.14 | 20.49 |  | 45.96 | 20.40 | 2.69 | 9.93 |
