# Supplementary material for: Comparative analysis of the secretomes of Schizophyllum commune and other wood-decay basidiomycetes during solid-state fermentation reveals its unique lignocellulose-degrading enzyme system
Source: Biotechnol Biofuels. 2016 Feb 20;9:42. doi: 10.1186/s13068-016-0461-x (PMC4761152; doi:10.1186/s13068-016-0461-x)
Supplement: Supplementary file 4 — 10.1186/s13104-016-1932-7 Schematic representation of nanoLC-MS/MS analysis of the extracellular proteins. [file 13068_2016_461_MOESM4_ESM.docx]

Reduction/Alkylation

In-gel trypsin digestion

SDS-PAGE

LC-MS/MS

Extracellular protein extraction

Database searching and data analysis

1

2

3

4

5

6

Six fractions


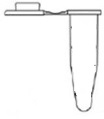

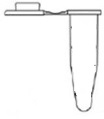

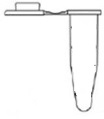

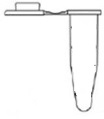

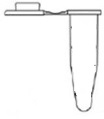

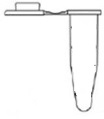


Small pieces(1 mm^2^)
